# Sperm: seminal fluid interactions and the adjustment of sperm quality in relation to female attractiveness

**DOI:** 10.1098/rspb.2009.0807

**Published:** 2009-07-08

**Authors:** Charlie K. Cornwallis, Emily A. O'Connor

**Affiliations:** 1 Edward Grey Institute, Department of Zoology, University of Oxford, Oxford OX1 3PS, UK; 2 Royal Veterinary College, Hawkshead Lane, Hatfield AL9 7TA, UK

**Keywords:** sexual selection, sperm competition, reproductive strategies, sperm quality, seminal fluid, female ornamentation

## Abstract

An important predictor of male fitness is the fertilizing efficiency of their ejaculates. Ejaculates are costly to produce and males are predicted to devote greater resources to copulations with reproductively superior females. It is well established that males allocate different numbers of sperm to ejaculates. However, less is known about how males adjust their sperm quality, which has important implications for our understanding of fertilization and the evolution of sexual strategies. Here we test in the fowl, *Gallus gallus*, whether males adjust their sperm velocity by differentially allocating seminal fluid to copulations with attractive and unattractive females. To disentangle the contributions of sperm and seminal fluid to sperm velocity, we separated and remixed sperm and seminal fluid from ejaculates allocated to females of different attractiveness. We show that dominant males increase the velocity of the sperm they invest in more attractive females by allocating larger ejaculates that contain seminal fluid that increases sperm velocity. Furthermore, we find weak evidence that males also allocate sperm with higher velocity, irrespective of seminal fluid, to more attractive females.

## 1. Introduction

Identifying the causes and consequences of variation in reproductive success is central to understanding the evolution of sexual strategies (Andersson 1994; Jennions *et al*. 2001). An important process determining variation in reproductive success is inter-sexual selection that occurs through mate choice and the differential investment of resources in sexual partners (Burley 1977; Bateson 1983; Sheldon 2000). Females are typically more discriminatory when choosing sexual partners, but under certain conditions males are also expected to be selective in their choice of mates (Parker 1983; Johnstone *et al*. 1996; Kokko & Monaghan 2001). Male choice is predicted to evolve when females vary in their ability to produce offspring, when males incur mating and/or parental costs and when the copulation opportunities males gain exceed the number of eggs they can fertilize (Parker 1983; Johnstone *et al*. 1996; Kokko & Monaghan 2001). These conditions are met when males have access to multiple sexual partners, such as in promiscuous mating systems, and their reproductive success is restricted by the costly production of ejaculates (Dewsbury 1982; Nakatsuru & Kramer 1982; Pitnick 1996; Olsson *et al*. 1997; Preston *et al*. 2001). Limited resources of semen, in combination with variation in female reproductive quality, are predicted to favour the evolution of strategic sperm allocation for reproductively superior females, which has been termed cryptic male choice (Parker 1998; Reinhold *et al*. 2002; Wedell *et al*. 2002).

There is strong empirical support for males allocating greater numbers of sperm to females that offer the highest reproductive benefits across a wide range of taxa (Wedell *et al*. 2002). For example, in insects, crustaceans, fish, birds and mammals, it has been shown that males allocate more sperm to larger or more ornamented females (Baker & Bellis 1993; Hunter *et al*. 2000; Bonduriansky 2001; Pilastro *et al*. 2002; Rubolini *et al*. 2006; Cornwallis & Birkhead 2007*b*; Sato & Goshima 2007). In addition, the extent to which sperm investment is biased towards favoured females can vary between males in relation to the frequency of copulation opportunities and risks of sperm depletion they face (Parker 1983; Shapiro *et al*. 1994; Preston *et al*. 2001; Montrose *et al*. 2008). This can result in males in favoured mating roles, such as socially dominant positions, being more prudent in their sperm allocation than males in disfavoured mating roles (Parker 1983, 1998; Hardling *et al*. 2008). Although it is well established that males adjust the number of sperm they ejaculate according to their social status and female attractiveness less is known about how males may promote their fertilization success through adjusting the fertilizing ability of their sperm (sperm quality, see Snook 2005 for further discussion). In humans (Kilgallon & Simmons 2005), Arctic charr, *Salvelinus alpinus*, (Rudolfsen *et al*. 2006), crickets, *Teleogryllus oceanicus*, (Simmons *et al*. 2007; Thomas & Simmons 2007) and the fowl, *Gallus gallus* (Cornwallis & Birkhead 2007*a*), it has now been shown that males strategically alter the quality of their sperm. However, despite the importance for understanding the processes determining variation in fertilization success the mechanisms underlying the adjustment of sperm quality remain unknown.

Theoretically, males may adjust the fertilizing efficiency of their sperm via two non-mutually exclusive mechanisms: (i) directly, by allocating sperm of different quality to ejaculates and/or (ii) indirectly, by allocating non-sperm components (seminal fluid) to ejaculates that in turn influence sperm performance by changing the resources available to sperm and the environmental conditions sperm experience (Poiani 2006). In a number of species, including the fowl, seminal fluid has been shown to contain a complex mixture of molecules that are costly to produce and that influence sperm performance (Lake 1984; Fujihara 1992). Males can become exhausted of seminal fluid even when ample sperm are available for ejaculation and therefore males are predicted to allocate seminal fluid according to the reproductive benefits they gain from copulations (Lefevre & Jonsson 1962; Cameron *et al*. 2007; Wigby *et al*. 2009). However, empirical evidence of whether males adjust the seminal fluid they allocate to ejaculates and whether this influences the fertilizing efficiency of sperm is lacking (see Wigby *et al*. 2009).

The aim of this study was therefore to experimentally test whether males adjust the quality of sperm they invest in attractive and unattractive females by strategically allocating seminal fluid to ejaculates. We tested these ideas in the fowl where it has previously been shown that males adjust their sperm swimming velocity, a predictor of fertilization success (Wishart & Palmer 1986; Froman *et al*. 2002), in relation to female attractiveness (Cornwallis & Birkhead 2007*a*).

The fowl live in small groups where male social status facilitates access to females; dominant males have higher copulation success than subordinate males (Pizzari *et al*. 2002). Promiscuity is common and males, particularly dominants, can become depleted of both sperm and seminal fluid (Pizzari *et al*. 2003). Limited semen reserves and the disparity between the copulation rates of dominant and subordinate males are thought to underlie the status-specific allocation of sperm numbers and the adjustment of sperm velocity according to female attractiveness (Cornwallis & Birkhead 2007*a*). Female attractiveness is determined by the expression of a sexual ornament, the comb, which is phenotypically and genetically correlated to the number and mass of eggs females lay (Cornwallis & Birkhead 2007*b*; Wright *et al*. 2007).

In this study, natural ejaculates were collected from dominant and subordinate males after copulations with attractive and unattractive females. We separated then remixed sperm and seminal fluid from ejaculates allocated to attractive and unattractive females to test the following predictions. (i) If males adjust their sperm velocity through the allocation of seminal fluid then: (a) the velocity of sperm invested in females with large combs will be reduced by seminal fluid allocated to females with small combs, and (b) the velocity of sperm invested in females with small combs will be increased by seminal fluid allocated to females with large combs. (ii) If males adjust their sperm velocity by investing sperm of different qualities in attractive and unattractive females, then mixing sperm with seminal fluid allocated to a female with a different comb size will not change sperm velocity. (iii) As dominant males bias their ejaculate investment towards attractive females more than subordinate males (Cornwallis & Birkhead 2006), the effect of seminal fluid on sperm velocity and/or differences in the quality of sperm allocated to attractive and unattractive females will be more pronounced in dominant compared with subordinate males.

## 2. Material and methods

### (a) Study population

We studied a population of fowl that are morphologically and behaviourally similar to its wild ancestor the red junglefowl, *G. gallus*, at the Tovetorp Zoological Research Station, University of Stockholm, during May–July 2007. All birds used were fully habituated to human presence. Two weeks prior to the start of the experiment, males (*n* = 30) were randomly assigned to pairs and placed in aviaries (6 × 6 m). Male social hierarchies were determined by observing aggressive interactions, in which all pairs were clear and stable with aggression being unidirectional. Females (*n* = 40) were kept in aviaries (6 × 6 m) in groups of three to eight individuals and every 10 days the size of their combs were measured from a digital photograph using Adobe Photoshop (see Cornwallis & Birkhead 2007*b* for more details). All males were kept separately from females to ensure they were sexually rested before each trial (no ejaculations for 48 hours; see Etches 1996).

### (b) Experimental design

The experiment involved four steps. (i) Males were presented with two females, one with a large comb and one with a small comb, and allowed to successively copulate in an alternate order with each female. (ii) Each ejaculate was collected and the seminal fluid was separated from the sperm. (iii) Sperm velocity was measured in seminal fluid from the same ejaculate to ascertain baseline patterns of sperm velocity. (iv) Sperm velocity was measured in seminal fluid from ejaculates allocated to females with the opposite comb size that were adjacent in copulation order. This was designed to test how seminal fluid allocated to more or less attractive females influenced sperm velocity. For each male, the experimental procedure was repeated on two separate occasions at least 48 h apart.

#### (i) Ejaculate collection

Males were temporally isolated from their pair male 15 min before being presented with a pair of females to prevent any interference during copulations. Previous work has shown that separating males for 15 min does not affect social hierarchies or lead to changes in status specific behaviour (Cornwallis & Birkhead 2008). Female pairs consisted of one female with a large comb and one female with a small comb and the difference in comb size was standardized across pairs (mean difference ± s.e.: 143 ± 12 mm^2^). The difference in comb sizes was within the range found within naturally free-ranging groups and has previously been shown to elicit changes in male sperm allocation patterns (Cornwallis & Birkhead 2007*b*). Each female was fitted with a plastic harness that covers the cloaca and allows the collection of natural ejaculates without contact with the female reproductive tract (Pizzari *et al*. 2003). Females were manually held with their heads pointed forwards for 1 min to allow the male to inspect the females. After 1 min, females were switched to a soliciting position and males were allowed to copulate. The first female the male copulated with was taken to be his choice of mate. Following the first copulation, females were re-presented but with wire netting placed over the female the male had just copulated with. This ensured the male could only copulate with the other female. This procedure was repeated until the male did not copulate for 15 min. This resulted in males copulating alternately with each female over a series of successive copulations. Ejaculates were collected after each copulation and the volume measured using a Gilson pipette (Pizzari *et al*. 2003). Males copulated up to six times, but on average copulated 4 ± 0.3 (mean±s.e.) times.

#### (ii) Sperm analysis

Ejaculates were homogenized by gentle shaking and 5 µl of semen were removed from the sample and stored in a water bath at 41°C (body temperature of the fowl (Etches 1996)). The remaining ejaculate was centrifuged for 1 min at 10 062*g* which separates sperm from seminal fluid (Mohan *et al*. 1995) and 10.5 µl of seminal fluid was removed from the top of the sample. To check whether seminal fluid was contaminated with sperm, 0.5 µl was examined on a slide under the microscope. Seminal fluid samples that still contained some sperm were not used (9% of cases). Two solutions for each ejaculate were created by adding sperm to: (i) seminal fluid isolated from the same ejaculate and (ii) seminal fluid from ejaculates allocated to the other female and adjacent in copulation order. Sperm (*ca* 0.5 µl) were added to each fluid to a concentration of approximately 10 × 10^6^ sperm ml^−1^ and mixed by gentle shaking. The sperm added to each solution contained some seminal fluid from the original ejaculate as certain seminal fluid proteins can bind to sperm (Töpfer-Petersen 1999) and it is difficult to remove all fluid from sperm. However, this was minimized with only a very small amount of seminal fluid from the original sample entering solutions in comparison to the amount of seminal fluid that sperm were added to (in excess of 20 times: *ca* greater than 0.5–10 µl) and this was the same across all ejaculates. After sperm were mixed with seminal fluid, samples were incubated in a waterbath at 41°C (the body temperature of fowl) for 3 min, which has previously been shown to be long enough to cause changes in measures of sperm quality (Mohan *et al*. 1995; M. G. Gillingham, C. K. Cornwallis & T. Pizzari 2005, unpublished data). Five microlitres of solution were placed on a microscope slide on a heated microscope stage at 41°C and recorded with a Basler A312fc digital video camera at 50 frames s^−1^ connected to a Nikon E200 microscope (Nikon Instruments Inc.) under negative phase contrast at ×100 magnification. The order in which sperm allocated to females with large and small combs were assayed was randomized. The velocity of individual sperm was measured using a computer-assisted sperm analysis system (Sperm Class Analyzer: SCA v. 3.0.3). Two fields per microscope slide and two microscope slides per sample were analysed (mean ± s.e. number of sperm tracked per sample = 513 ± 41.3). All sperm that had a forward movement over 5 µs^−1^ for 20 frames were measured and the median average path velocity (VAP µm s^−1^) was calculated for each sample from the four recordings. Median VAP was highly correlated with mean VAP (Pearson's correlation coefficient: *R* = 0.98), but the median was used to ensure that for all ejaculates measures of central tendency were not influenced by non-normal distributions of sperm velocity. Various measures of sperm velocity can be calculated such as straight line velocity (VSL) and curvilinear velocity (VCL), but we used VAP because it has been shown to correlate to fertilization success in the fowl (Wishart & Palmer 1986) and was highly correlated with VSL and VCL (VAP versus VSL: *R* = 0.96. VAP versus VCL: *R* = 0.97).

### (c) Statistical analysis

Four analyses were conducted. (i) Variation in male mate choice was analysed using a generalized linear mixed model (GLMM) with a binary error distribution (1 = chosen, 0 = not chosen). Male social status (dominant, subordinate) and female comb size (large, small) were entered as fixed factors. Because a male's choice for one female determines choice for the other female, only data from one randomly chosen female per male were analyzed. (ii) Variation in ejaculate volume over successive copulations was analysed using a GLMM with restricted maximum-likelihood estimation (REML). Ejaculate volume was positively skewed, but defining the model with a lognormal error distribution generated normal residuals and homogeneous variance. We examined ejaculate volume across pairs of ejaculates, which we refer to as ‘ejaculate pair order’ (1 = ejaculates 1 + 2, 2 = ejaculates 3 + 4, 3 = ejaculates 5 + 6). This was done because the experimental design swapped sperm and seminal fluid from ejaculates adjacent in order in the copulation series and therefore the unit of experimentation was pairs of ejaculates. Male social status and female comb size were entered as fixed factors and ejaculate pair order was entered as a covariate. (iii) We analysed variation in sperm velocity measured in seminal fluid from the same ejaculate using a GLMM with a normal error distribution and REML estimation. Male social status and female comb size were entered as fixed factors and ejaculate pair order and ejaculate volume were entered as covariates. (iv) Variation in the change in sperm velocity (sperm velocity measured in seminal fluid allocated to the female with the opposite comb size—sperm velocity measured in seminal fluid from the same ejaculate) was analysed using a GLMM with a normal error distribution and REML estimation. Male social status and female comb size were entered as fixed factors and ejaculate pair order, the volume of the ejaculate sperm originated from and the volume of the ejaculate fluid came from were entered as covariates. In all models, replicate, group and male nested within group were entered as random factors, which took account of the non-independence of data arising from measurements made on ejaculates from the same male, from males being in the same groups and measurements made during the same replicate (Littell *et al*. 2006).

Analyses were performed in SAS v. 9.2 (Littell *et al*. 2006). The significance of fixed effects (factors and covariates) in GLMMs were examined using Wald type adjusted *F*-statistics and the effect with the highest *p*-value was sequentially dropped until only significant terms (*p* < 0.05) remained in the model (Crawley 2002). The Kenward & Roger (1997) method for calculating denominator degrees of freedom was used, which is specifically designed for analysing unbalanced repeated measures data with models that contain multiple random effects (Kenward & Roger 1997; Littell *et al*. 2006). The significance of random effects was assessed using log-likelihood ratio tests (Self & Liang 1987). Details of all analyses are provided in tables in the electronic supplementary material.

## 3. Results

### (a) Male mate choice and adjustment of ejaculate volume

**Figure 1.**
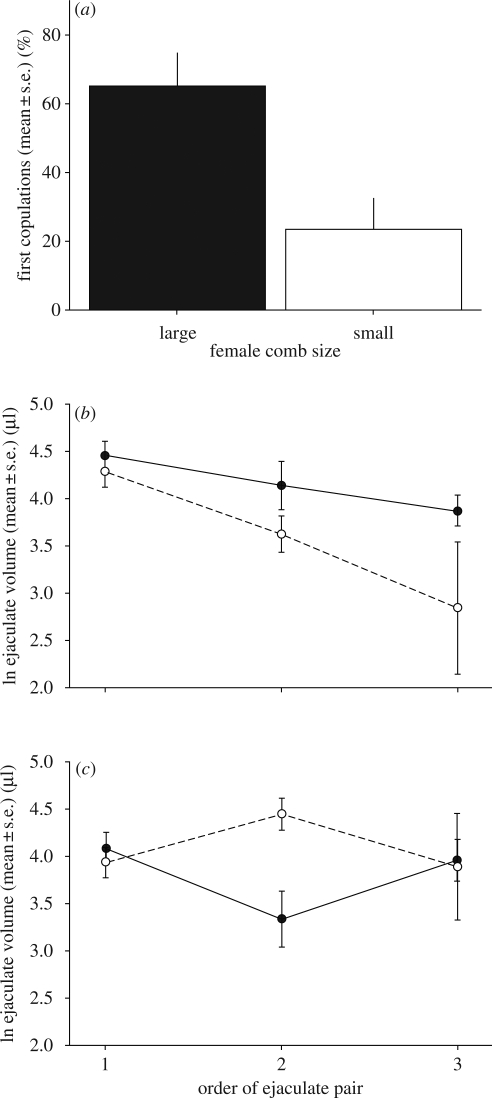
Male mate choice and the ejaculate volume males of different status allocated to females with large and small combs over successive copulations. (*a*) Dominant and subordinate males preferred to copulate with females with large combs (electronic supplementary material, table S1; comb size: *p* = 0.01). As mate choice for one female automatically means the other female is not chosen (non-independent data), only data from one randomly chosen female per male is plotted. Error bars represent variation across males in their average choice for females. (*b*) Dominant males allocated larger ejaculates to females with large combs relative to females with small combs and this difference became increasingly pronounced over successive copulations (electronic supplementary material, table S2; status * comb size * ejaculation order: *p* = 0.01). (*c*) In contrast, subordinate males allocated ejaculates of similar size to both females (Table S2. Status * comb size * ejaculation order: *p* = 0.01). Black dots and solid lines represent females with large combs and white dots and dashed lines represent females with small combs.

Consistent with previous research, we found that males preferred to copulate with females with large combs (figure 1*a*; electronic supplementary material, table S1; comb size: *F*_1,20_ = 7.71, *p* = 0.01). The volume of ejaculates males produced declined over successive copulations (electronic supplementary material, table S2; ejaculate pair order: *F*_1,136_ = 25.75, *p* < 0.0001). However, dominant males allocated relatively larger ejaculates to females with large combs, which became more pronounced over successive ejaculations (figure 1*b*), whereas subordinate males allocated ejaculates of similar size to both females (figure 1*c*; electronic supplementary material, table S2; status * ejaculate pair order * comb size: *F*_1,113_ = 6.57, *p* = 0.01).

### (b) Sperm velocity measured in seminal fluid from the same ejaculate

**Figure 2.**
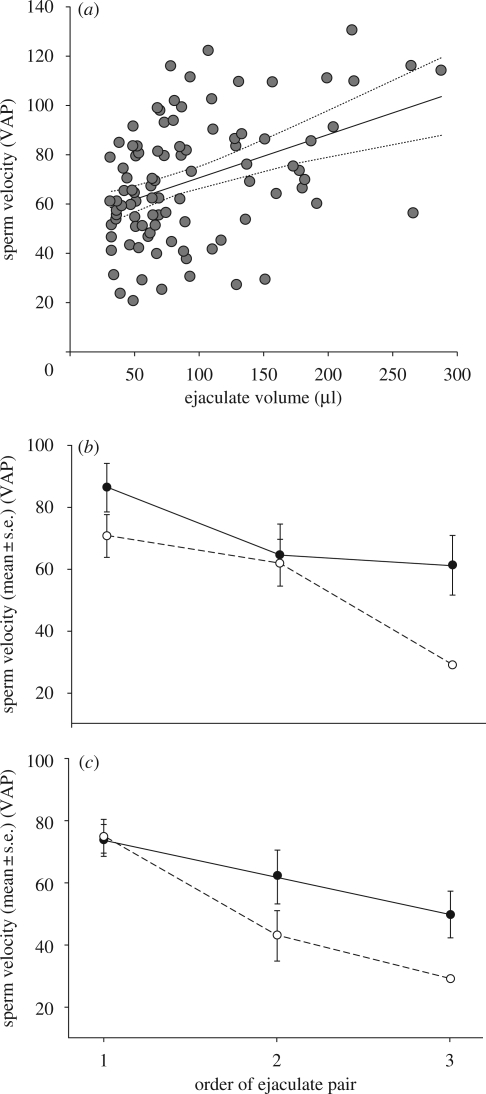
Sperm velocity when measured in seminal fluid from the same ejaculate. (*a*) Sperm from larger ejaculates had higher velocity (electronic supplementary material, table S3: *p* = 0.01). Points are individual ejaculates and the line represents the relationship predicted by the GLMM with 95 per cent confidence intervals. (*b*) Dominant males’ sperm velocity declined over successive ejaculations with females with small combs, whereas sperm from ejaculates allocated to females with large combs had relatively higher velocity (electronic supplementary material, table S3; status * comb size * ejaculation order: *p* = 0.04). (*c*) Subordinate males’ sperm velocity declined with both large and small combed females, but the decrease was more pronounced in sperm from ejaculates allocated to females with small combs (electronic supplementary material, table S3; status * comb size * ejaculation order: *p* = 0.04). Black dots and solid lines represent females with large combs and white dots and dashed lines represent females with small combs.

There was a strong relationship between sperm velocity and the volume of the ejaculate sperm came from (figure 2*a*; electronic supplementary material, table S3; ejaculate volume: *F*_1,90_ = 14.61, *p* = 0.0002). Sperm velocity also declined over successive copulations (electronic supplementary material, table S3; ejaculate pair order: *F*_1,93_ = 7.04, *p* = 0.009), but the rate of decrease was dependent upon the status of the copulating male and the comb size of the female (electronic supplementary material, table S3; status * ejaculate pair order * comb size: *F*_1,82_ = 4.41, *p* = 0.04). Dominant males allocated ejaculates with sperm of higher velocity to females with large combs across the majority of their copulations (figure 2*b*). In contrast, subordinate males allocated ejaculates that contained sperm of similar velocity to both females during initial copulations and only in subsequent copulations invested ejaculates with higher velocity sperm in females with large combs (figure 2*c*).

### (c) Changes in sperm velocity caused by seminal fluid

**Figure 3.**
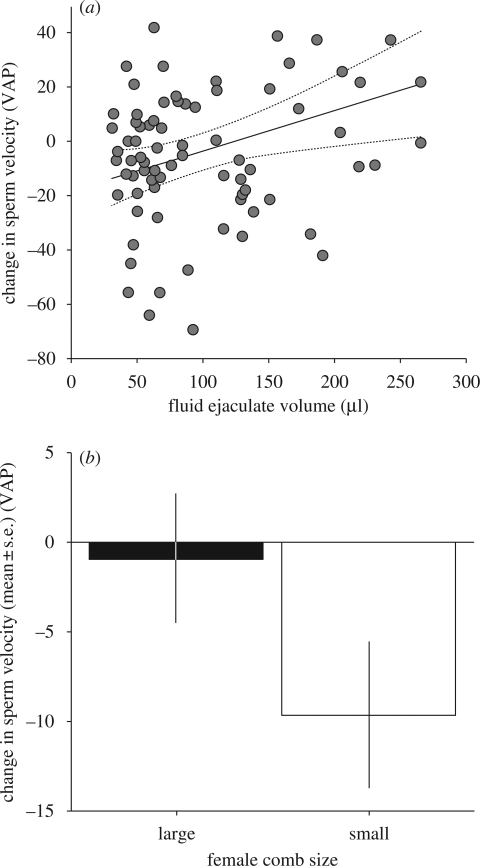
The change in velocity when sperm were measured in seminal fluid allocated to a female with a different comb size (change = sperm velocity measured in seminal fluid allocated to a female with an opposite comb size−sperm velocity measured in seminal fluid from the same ejaculate). (*a*) The change in sperm velocity was positively related to the volume of the ejaculate that the seminal fluid was taken from (electronic supplementary material, table S4; fluid ejaculate volume: *p* = 0.04). Points are individual ejaculates and the line represents the relationship predicted by the GLMM with 95 per cent confidence intervals. (*b*) The velocity of sperm from ejaculates allocated to females with large combs did not change when mixed with seminal fluid ejaculated with females with small combs (electronic supplementary material, table S4; change in velocity versus 0: *t* = 0.33, *p* = 0.74), whereas the velocity of sperm from ejaculates allocated to females with small combs was reduced by seminal fluid ejaculated with large combed females (electronic supplementary material, table S4; change in velocity versus 0: *t* = −2.44, *p* = 0.02).

After controlling for the effects of the volume of the ejaculate sperm were taken from (electronic supplementary material, table S4; sperm ejaculate volume: *F*_1,69_ = 5.03, *p* = 0.02), the ejaculate volume from which seminal fluid originated had a positive effect on sperm velocity (figure 3*a*; electronic supplementary material, table S4; fluid ejaculate volume: *F*_1,66_ = 4.28, *p* = 0.04). This meant that if sperm were mixed with seminal fluid taken from a large ejaculate their velocity increased, whereas if sperm were mixed with seminal fluid from a small ejaculate their velocity decreased (figure 3*a*). In addition, after controlling for the effects of ejaculate volume, there was a tendency for sperm allocated to females with large and small combs to react differently to their seminal fluid environment (figure 3*b*; electronic supplementary material, table S4; comb size: *F*_1,69_ = 3.25, *p* = 0.07). The velocity of sperm allocated to females with large combs was not influenced by seminal fluid allocated to females with small combs (figure 3*b*; electronic supplementary material, table S4; *t*-test: 1.42 ± 4.25 versus 0, *t* = 0.33, *p* = 0.74). However, the velocity of sperm invested in females with small combs was reduced by seminal fluid allocated to females with large combs (figure 3*b*; electronic supplementary material, table S4; *t*-test: −8.77 ± 3.60 versus 0, *t* = −2.44, *p* = 0.02). This suggests that there were differences in the sperm allocated to attractive and unattractive females that led to different interactions with seminal fluid.

## 4. Discussion

Evidence from insects, fish, birds and humans has illustrated that males are able to strategically adjust the quality of the sperm they invest in females, but the mechanisms by which males do this have not previously been investigated (Kilgallon & Simmons 2005; Rudolfsen *et al*. 2006; Cornwallis & Birkhead 2007*a*; Thomas & Simmons 2007). The aim of this study was to test whether males adjust their sperm velocity by differentially allocating seminal fluid to attractive and unattractive females. We show that the adjustment of sperm velocity in response to variation in female attractiveness was due to: (i) dominant males allocating larger ejaculates to attractive females that contained seminal fluid which increased sperm velocity and (ii) males investing sperm in attractive and unattractive females that reacted differently to the seminal fluid environment, although these effects were weak. We discuss how these findings may aid our understanding of the physiological basis to variation in fertility and the evolution of sexual strategies.

### (a) Ejaculate volume and sperm velocity

The main result from this study showed that sperm velocity was increased by a fixed volume of seminal fluid from larger ejaculates. Previous research has demonstrated positive correlations between different ejaculate parameters (Malo *et al*. 2005; Snook 2005; Gomendio *et al*. 2007) and our results suggest that to some extent these relationships may be driven by the effects of seminal fluid. Furthermore, this study has demonstrated that this mechanism is utilized in a functional context with males adjusting the velocity of sperm they invested in females of different attractiveness through the allocation of seminal fluid. The correspondence between changes in sperm velocity and ejaculate volume was, however, different for dominant and subordinate males, suggesting that factors linked to social status may influence seminal fluid composition in addition to ejaculate volume. It is unknown how the composition of seminal fluid from males of different social status changes with ejaculate size and which components are responsible for increasing sperm velocity. Nevertheless, there has been a substantial amount of research on how the chemical environment created by the male reproductive tract and by seminal fluid influences measures of sperm quality, particularly in mammals and some insects (Poiani 2006). Much less is known about birds, but in the fowl seminal fluid is made up of two main components, seminal plasma and transparent fluid. Seminal plasma is derived from the testes and excurrent ducts and is mixed with sperm as they travel down the vas deferens. Transparent fluid originates from lymphatic folds around the cloaca and is added to sperm upon ejaculation (Lake 1984; Fujihara 1992; Etches 1996). Both fluids contain a complex cocktail of chemicals that have been shown to have a diversity of effects on sperm including the stimulation of motility and metabolism (Terada 1980; Ashizawa & Okauchi 1984; Lake 1984; Fujihara 1992; Froman 2003). Proteins, glutamate and Ca^+^ in seminal plasma have been shown to mediate sperm motility causing stimulatory and inhibitory effects (Mohan *et al*. 1995; Froman 2003). Transparent fluid can increase sperm velocity by creating an alkaline environment and by providing glucose and aldose, which are metabolized by sperm to generate ATP (Nishiyama & Fujishima 1961; Wishart & Palmer 1986). The amounts of particular compounds that are added to ejaculates during copulation are likely to be influenced by the strength of ejaculatory contractions (Lake 1957), which may provide an explanation for the positive link between ejaculate size and sperm velocity.

In addition to the effects of ejaculate volume, sperm velocity was influenced by ejaculation order which is in line with previous findings (Birkhead *et al*. 1995; Cornwallis & Birkhead 2007*a*). The decline in sperm velocity was dependent upon male social status and female comb size; dominant males allocated ejaculates with higher velocity sperm to attractive females across the majority of their copulations whereas subordinate males only allocated ejaculates with higher velocity sperm to attractive females after initial copulations. The mechanisms causing these differences are unknown. However, we present one possible explanation that requires further testing. Males may alter the velocity of sperm they allocate to copulations by strategically firing their left and right ejaculatory ducts, which can operate independently (Nishiyama 1950; Lake 1957). If one duct is more likely to fire than the other, and the probability of both ducts firing is dependent on how stimulated males are during copulations, then sperm from one duct may always contribute to ejaculates whereas sperm from both ducts will only be ejaculated when males are more stimulated, for example, when copulating with attractive females. Stratification of sperm occurs within the ductus deferens and sperm velocity increases as they migrate closer to the cloaca (S. Lupold, C. K. Cornwallis & T. R. Birkhead 2006, unpublished data), which may explain the strong negative effect of copulation order on sperm velocity found in this and other studies (Birkhead *et al*. 1995; Koldras *et al*. 1996). Stratification of sperm within the ductus deferens in combination with the probability of ejaculatory ducts firing being dependent upon female attractiveness may lead to females with large combs receiving sperm from both ducts, but less attractive females only getting sperm from the duct that fires more frequently and thus sperm of lower velocity. It is often observed in birds, including the fowl, that one testis is larger than the other (Friedmann 1927; Lake 1984), which may be linked to different rates at which sperm are used from the two ejaculatory ducts. Furthermore, differential firing of left and right ejaculatory ducts may contribute to how males strategically change the number of sperm in their ejaculates, a phenomenon that is widespread but for which the mechanism remains unknown (Wedell *et al*. 2002).

### (b) Sperm: seminal fluid interactions

The final results presented in this study suggest that sperm allocated to attractive and unattractive females may differ in how they respond to seminal fluid. The velocity of sperm from ejaculates invested in attractive females remained consistent across seminal fluid treatments, whereas the velocity of sperm from ejaculates invested in unattractive females was reduced by seminal fluid allocated to attractive females. These results are quite different from our original prediction that sperm invested in unattractive females would be increased by seminal fluid allocated to attractive females and *vice versa*. However, it suggests that there are differences between sperm from ejaculates invested in attractive and unattractive females with the velocity of sperm invested in unattractive females being more sensitive to the effects of seminal fluid. It has previously been shown that transparent fluid can negatively affect sperm motility when sperm are in low concentrations (Nishiyama *et al*. 1971). Less attractive females receive ejaculates with fewer sperm (Pizzari *et al*. 2003) and this may render them more susceptible to the adverse effects of transparent fluid (Lake 1984; Mohan *et al*. 1995; Etches 1996). Selection may therefore favour the evolution of allocation strategies whereby the seminal fluid added to ejaculates is adjusted according to the number of sperm inseminated.

Irrespective of the physiological basis by which males alter the sperm and seminal fluid in their ejaculates our results have a number of implications for the evolution of reproductive strategies. It is evident that sperm performance is not solely an attribute of sperm, but is determined by interactions with seminal fluid, which are likely to have important effects on fertilization success. Variation in fertilization success caused by differential interactions between sperm and seminal fluid allocated to females of varying attractiveness is likely to shape the evolution of male allocation strategies (Poiani 2006; Cameron *et al*. 2007; Wigby *et al*. 2009). It has previously been thought that males adjust the size of their ejaculates according to the reproductive benefits available from copulations because of the fertilization advantage gained by inseminating more sperm (Wedell *et al*. 2002). However, the results of this study suggest that the relationship between ejaculate size and paternity may be driven not only by larger ejaculates having more sperm, but also by sperm of higher velocity. The evolution of the strategic adjustment of ejaculate size may therefore be shaped by the effects of sperm velocity as well as sperm number on fertilization success. Furthermore, as males adjusted their sperm velocity according to female comb size, these results have implications for the evolution of female phenotypes. Females with larger combs secured bigger ejaculates containing higher velocity sperm, particularly from dominant males that are preferred by females (Parker & Ligon 2003). This in turn may generate directional sexual selection for further exaggeration of female ornamentation. Finally, these results add to recent theoretical and empirical work that has highlighted the importance of considering the effects of both seminal fluid and sperm on fertilization success when trying to understand the evolution of ejaculate composition and allocation strategies (Cameron *et al*. 2007; Wigby *et al*. 2009). Further experimentation is now needed to reveal how males adjust the sperm and seminal fluid in their ejaculates and to quantify the outcome of interactions between sperm and seminal fluid *in vivo*.
